# Hungarian Validation of the Acceptance of Illness Scale Among Cancer Patients

**DOI:** 10.1192/j.eurpsy.2026.10655

**Published:** 2026-07-31

**Authors:** D. Őri, R. Chowdhry, L. Losonczy, E. Szenczi-Pinter, A. Guttengeber, A. Petranyi, V. K. Bognar, E. Foldesi, G. Purebl, A. Kegye

**Affiliations:** 1 Institute of Behavioural Sciences, Semmelweis University; 2 Department of Mental Health, Heim Pal National Pediatric Institute, Budapest, Hungary; 3 San Francisco School of Medicine, University of California, San Francisco, United States; 4 Department of Psychiatry and Psychotherapy; 5 Department of Clinical Psychology; 6 Department of of Internal Medicine and Hematology; 7 Department of Urology; 8 Institute of Pancreatic Diseases, Semmelweis University, Budapest, Hungary

## Abstract

**Introduction:**

The Acceptance of Illness Scale (AIS) is a widely used questionnaire developed to assess illness acceptance in various conditions, including cancer. The AIS measures the extent to which individuals are able to accept their illness and adapt to the limitations and challenges it imposes on their daily lives. Its latent factor structure has been explored in a few countries; however, the psychometric properties of the scale have not yet been investigated in Hungary.

**Objectives:**

We aimed to explore the factor structure of the AIS and examine the key psychometric properties of the Hungarian version.

**Methods:**

A paper-based survey was administered to patients at the clinics of Semmelweis University, Hungary. The AIS is an 8-item self-report measure with a single subscale. Exploratory and confirmatory factor analyses were conducted to examine its factor structure, and Cronbach’s alpha coefficient was calculated to assess internal consistency. The *helplessness/hopelessness* and *fighting spirit* subscales of the Mini-Mental Adjustment to Cancer scale (Mini-MAC) were used to evaluate convergent and divergent validity. Intraclass correlation coefficients will be applied to assess test–retest reliability.

**Results:**

This is ongoing research. To date, 259 participants have completed the questionnaire, and data collection is still in progress. Parallel analysis supported a single-factor solution, indicating a unidimensional structure on which all eight items loaded. The root mean square error of approximation (RMSEA) was acceptable (0.068), and the incremental fit indices [comparative fit index (CFI) = 0.996, Tucker–Lewis index (TLI) = 0.993] exceeded the commonly accepted threshold of 0.90. Cronbach’s alpha was 0.885, which indicated good internal consistency. Convergent and divergent validity with the Mini-MAC subscales showed significant correlations: *fighting spirit* (r = 0.204, p = 0.001) and *helplessness/hopelessness* (r = –0.510, p < 0.0001). Test–retest reliability analyses are currently underway.

**Conclusions:**

Preliminary findings support a unidimensional structure of the AIS with good internal consistency. Further validity and reliability analyses will be completed in the coming months, prior to poster presentation. We expect that the Hungarian version of the AIS will demonstrate suitable psychometric properties for use among individuals with physical health conditions, particularly cancer, in Hungary.

**Disclosure of Interest:**

None Declared

